# Emergency Pediatric Intubations in an Urban Children’s Hospital Before and After Just-in-Time Training for Video Laryngoscopy

**DOI:** 10.7759/cureus.19892

**Published:** 2021-11-25

**Authors:** Evan Lum, Sherri Sommer-Candelario, So Yung Choi, Stephanie Delos Santos, Kagen Aeby, Jannet Lee-Jayaram

**Affiliations:** 1 Pediatrics, University of Hawai'i, John A. Burns School of Medicine, Honolulu, USA; 2 Pediatric Transport, Kapiʻolani Medical Center for Women & Children, Honolulu, USA; 3 Quantitative Health Sciences, University of Hawai'i, John A. Burns School of Medicine, Honolulu, USA; 4 Pediatrics, Kapiʻolani Medical Center for Women & Children, Honolulu, USA

**Keywords:** laryngoscopy, healthcare simulation, just-in-time training, pediatric intubation, videolaryngoscopy

## Abstract

Objectives: The use of video laryngoscopy (VL) may augment emergency pediatric intubations outside the operating room (OR). Our objective was to describe the proportion of use and complications with VL before and after implementation of a VL just-in-time training (JITT).

Study design: This study was a retrospective chart review of pediatric intubations performed outside the OR at a single women and children’s hospital from January 2015 to March 2020. Data were collected on patient age, intubation method, operator characteristics, adverse events, number of attempts, condition leading to intubation, and hospital location. Data were separated into pre-JITT (January 1, 2015 to April 31, 2018) and post-JITT (May 1, 2018 to March 1, 2020) periods. Descriptive statistics were used comparing pre- and post-JITT periods for VL use, and the complications of intubations with multiple attempts (IMAs) and intubations with one or more adverse events (AEs).

Results: A total of 231 pediatric patients were intubated during the study period; 154 intubations in the pre-JITT and 77 intubations in the post-JITT periods. Pre- and post-JITT VL use was 17 (11%) and 17 (22%), respectively. With pre-JITT VL, there were four (23%) IMAs and zero (0%) intubation with one or more AE. With post-JITT VL, there were eight (47%) IMAs and one (6%) intubation with one or more AE.

Conclusion: The proportion of emergency pediatric intubations using VL increased after the institution of a JITT. There was no significant change in IMAs and AEs. The infrequency of pediatric intubations makes drawing conclusions regarding the impact on IMAs and AEs challenging. JITT may increase VL use for emergency pediatric intubations outside the OR and may be considered for refresher training, especially during the coronavirus disease 2019 (COVID-19) pandemic.

## Introduction

Critically ill and injured pediatric patients frequently require intubation during the course of their medical care. Pediatric patients present additional challenges due to their anatomical differences that make intubation more difficult including smaller mouth, relatively larger tongue, larger occiput, floppier epiglottis, and narrow airway [1,2]. Additionally, pediatric patients have a higher metabolic rate, less reserve, and faster onset of hypoxia, making first-pass intubation success an even more critical goal than with adult patients [1,2].

Video laryngoscopy (VL) is a method by which the glottic opening is visualized indirectly using a camera. The advantages of VL over direct laryngoscopy (DL) have been highlighted during the COVID-19 pandemic. Use of VL over DL for intubation is recommended to minimize risk by increasing first attempt success and distance from the patient’s airway during this potentially aerosol-generating procedure [3,4]. While VL in adults may reduce the number of failed intubations, improve the glottic view, and reduce laryngeal trauma, in children, some evidence suggests that VL may lead to prolonged intubation with an increased rate of intubation failure when compared to DL [5,6]. VL has not demonstrated a consistent reduction in the number of intubation attempts nor the number of complications associated with intubation. Nevertheless, in light of the coronavirus disease 2019 (COVID-19) pandemic, the need to upskill medical providers in the use of VL has become evident.

Training methods for initial skill development and maintenance of skill retention vary. Just-in-time training (JITT), which originated in the automotive industry, is a planned, on-the-job, short unit of instruction provided on-demand in close proximity to the need for the training [7]. JITT has demonstrated increased learner confidence in lumbar puncture, improved skill in VL and pediatric advanced life support, and decreased procedural skill decay in transvenous pacing catheter placement [8-11]. Its advantages include short time for training, in-situ location for training, and availability for repeated training.

JITT for VL was instituted at our tertiary care hospital in 2018 to upskill providers and staff in the use of this alternate method for visualizing the airway during intubations. The goal of this study was to describe the proportion of use and intubation complications with pediatric VL before and after implementation of VL JITT, prior to the COVID-19 pandemic. We excluded post-pandemic intubations in the expectation that the use of VL, the difficulty of intubations with additional personal protective equipment (PPE), and illness severity of patients would be affected by the COVID-19 pandemic.

## Materials and methods

**Figure 1:**
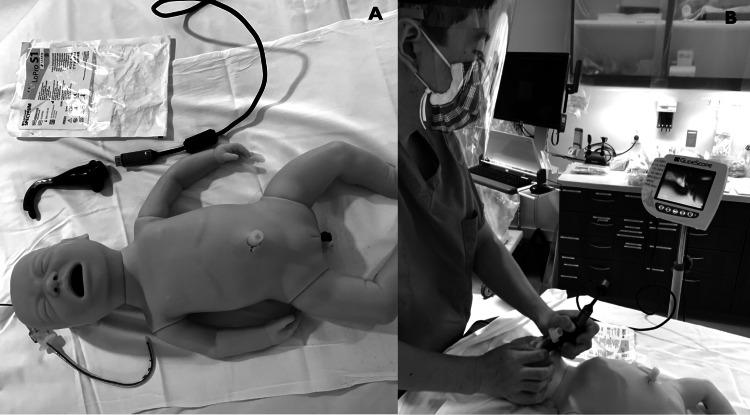
Just-in-time training equipment and hands-on practice. A: Infant manikin, video laryngoscopy blade, and a 3.5 endotracheal tube with a stylet. B: Hands-on practice during just-in-time training in the emergency department.

A retrospective electronic chart review was performed of all intubations outside the OR at a single, urban, tertiary care, women and children’s hospital in Honolulu, Hawaii from January 2015 (when VL was available at the institution) to March 2020 (when severe acute respiratory syndrome coronavirus 2 [SARS-CoV-2] infections were initially reported in Hawaii). The hospital is the only children’s specialty hospital in the state, with the next closest pediatric hospital 2,600 miles away, and serves as the referral center for pediatric patients throughout the Pacific basin. Intubations outside the controlled OR environment were the focus, as these are generally emergent in nature, not performed by anesthesiologists, and high risk due to urgency and unknown fasting status. Intubation procedure notes were reviewed and data were collected on patient age, intubation method, provider specialty, adverse events, number of attempts, condition leading to intubation, and hospital location. In May 2018, we implemented a 15-minute VL JITT with hands-on simulated practice for emergency department (ED) and pediatric intensive care unit (PICU) providers and staff during regular work hours. JITT was provided by an ED attending physician and the transport nursing manager when workflow and patient census in the ED or PICU allowed. Participants included resident physicians, physicians, nurses, and respiratory therapists. All staff who care for critically ill patients outside the OR were invited to participate, even those who do not primarily perform intubations. This was to increase all staff's familiarity with equipment, including setting up, and how to best support the provider intubating, thereby increasing the likelihood of VL use. Participation was voluntary and repeatable. The JITT started with orientation to the equipment, then VL intubation demonstration by the instructor, and finally guided participant hands-on practice intubating an infant manikin. Equipment included a low-fidelity, intubatable, infant manikin, a VL (GlideScope®, Verathon, Inc., Bothell, Washington), an endotracheal tube, and stylet (Figure 1).

Pediatric intubation data were analyzed as training was focused on pediatric providers. Pediatric patients were defined as patients younger than 18 years old at the time of intubation. The data were separated into pre-JITT (January 1, 2015 to April 31, 2018) and post-JITT (May 1, 2018 to March 1, 2020) periods. The Institutional Official of Hawaii Pacific Health determined that the study was not a research subject to be reviewed by the Institutional Review Board. Descriptive statistics were used comparing pre- and post-JITT periods for VL use and the complications of (1) intubations with multiple attempts (IMAs) and (2) intubations with one or more adverse events (AEs). Change in VL use pre- and post-JITT was tested using Fisher’s exact test.

## Results

**Figure 2:**
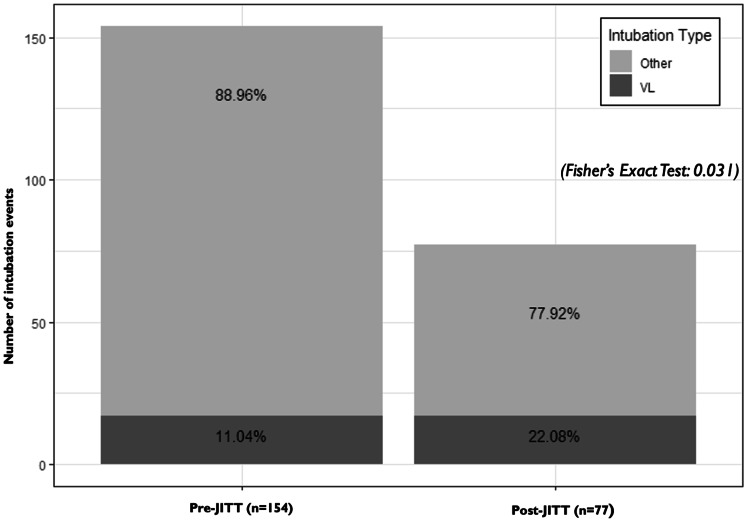
All intubations pre- and post-JITT. Pre-JITT: 154 intubations; VL use was 17 (11%). Post-JITT: 77 intubations; VL use 17 (22%). There was increased video laryngoscopy use from the pre- to post-JITT periods (p = 0.03). JITT, just-in-time training; VL = video laryngoscopy.

A total of 268 patients were intubated outside the OR during the study period; 231 were pediatric patients. VL was used in 34 intubations and VL was not used in 197. There were no significant differences with respect to resident involvement, patient medical condition, and patient age <1 year (Table 1). There were 154 intubations in the pre-JITT and 77 intubations in the post-JITT period. There was a significant difference in pre- and post-JITT VL use, which was 17 (11%) and 17 (22%), respectively (p = 0.03, Figure 2). ED and PICU intubations were further analyzed as JITT was conducted in these units. Pre-JITT pediatric intubations in the ED and PICU numbered 72 (47%) and 74 (48%) with 12 (17%) and five (7%) intubations using VL. Post-JITT pediatric intubations in the ED and PICU numbered 32 (42%) and 44 (57%), with 14 (44%) and three (7%) intubations using VL (Tables 2, 3). With pre-JITT VL, there were four (23%) IMAs and zero (0%) intubation with one or more AE. With post-JITT VL, there were eight (47%) IMAs and one (6%) intubation with one or more AE (Table 4).

**Table 1:** Characteristics of pediatric intubations (n = 231). ^1^Fisher's exact test; VL = video laryngoscopy.

Characteristics	No VL (n = 197)	VL (n = 34)	p-value^1^
Intubation inserted by, n (%)			0.384
Non-resident or unknown	177 (90%)	29 (85%)	
Resident	20 (10%)	5 (15%)	
Condition, n (%)			0.111
Cardiovascular	31 (15.7%)	3 (8.8%)	
Neurological	64 (32.5%)	17 (50.0%)	
Respiratory	57 (28.9%)	6 (17.6%)	
Sepsis/shock	26 (13.2%)	2 (5.9%)	
Other/unknown	19 (9.6%)	6 (17.6%)	
Age, n (%)			0.543
0 year	58 (29%)	8 (23.5%)	
1+ years	139 (71%)	26 (76.5%)	

**Table 2:** Use of VL among ED patients (n = 104). ^1^Fisher’s exact test; VL = video laryngoscopy; ED = emergency department.

	Pre (n = 72)	Post (n = 32)	p-value^1^
Other intubations	60 (83%)	18 (56%)	0.006
VL intubations	12 (17%)	14 (44%)	

**Table 3:** Use of VL among PICU patients (n = 118). ^1^Fisher’s exact test; VL = video laryngoscopy; PICU = pediatric intensive care unit.

	Pre (n = 74)	Post (n = 44)	p-value^1^
Other intubations	69 (93%)	41 (93%)	>0.999
VL intubations	5 (7%)	3 (7%)	

**Table 4:** Pediatric patients with the use of VL (n = 34). ^1^Fisher’s exact test; VL = video laryngoscopy; AEs = adverse events.

	Pre (n = 17)	Post (n = 17)	p-value^1^
Intubation attempts			0.148
1	6 (35%)	7 (41%)	
≥2	4 (24%)	8 (47%)	
Unknown	7 (41%)	2 (12%)	
AEs			>0.999
None	16 (94%)	15 (88%)	
1 or more	0 (0.0%)	1 (6%)	
Unknown	1 (6%)	1 (6%)	

## Discussion

VL use for pediatric intubations increased in the ED from the pre-JITT to the post-JITT period, but there was no significant change in the PICU. A systematic review and meta-analysis of adult and pediatric intubations outside the OR comparing VL to DL did not demonstrate any improvement in first-pass success in the ED with VL but did do so in the intensive care unit setting [12]. PICU intubations are more likely to be planned than ED intubations, after observation of clinical deterioration, which may influence the choice between DL and VL.

The infrequency of pediatric intubations with complications at this study’s institution makes drawing significant conclusions regarding the impact on IMAs and AEs challenging. A 2016 report from the National Emergency Airway Registry of pediatric intubations in the ED analyzed 1,053 intubation events [13]. The first attempt's success rate was 83% and AE was noted in 15% of all encounters. Documentation standards for the participating centers were established by the registry allowing for more accurate quantification. Documentation at this study’s institution was noted to be variable, with inconsistent reporting of intubation attempts and AE, making accurate quantification difficult. In the 2016 report, VL was a predictor of first attempt success over DL among participating institutions that did not include any children’s hospitals.

JITT at this study’s institution demonstrated potential success in increasing VL use for emergency pediatric intubations outside the OR but a decrease in complications was not observed. The total number of complications may have been too low to detect any statistically significant changes. While a meta-analysis completed in 2017 on the use of VL in pediatric intubations did not demonstrate any advantage of using VL, a more recently published study, completed before the start of the pandemic, found VL was associated with higher first-attempt success over DL in pediatric ED patients [14].

Given that VL is recommended for all intubations during the COVID-19 pandemic, further intensive training may be required for providers participating in emergency pediatric intubations outside the OR in this new era. Reports after the start of the COVID-19 pandemic have confirmed that VL use has increased in emergency intubations [15,16]. Brief JITT for intubation using DL in a PICU did not improve resident first attempt or overall success but did increase resident participation in intubation events [17]. VL training using simulation-based mastery learning has demonstrated improved skill in pediatric residents when tested on manikins and a post-training first attempt success rate of 77.8%, although pre-training first attempt success rate was not collected [18]. The resident training was conducted using mastery learning, requiring persistent training to a demonstrated competency level, and took between 30 minutes and two hours. While mastery learning is superior for skill acquisition in comparison to non-mastery simulation training, it also requires significantly more time [19]. As VL use increases by necessity, shorter, repeatable, JITT VL training may be more practical for busy pediatric healthcare providers.

In addition to necessity, there are other reasons that VL may be beneficial to use. During traditional DL, only the person intubating can visualize the airway, while the rest of the team is not able to determine the exact procedure step that is occurring. With VL, other providers and staff on the team can simultaneously visualize the airway and recordings can be preserved for future review. This concept of intubation sharing may be useful for coaching trainees, sharing information, research, and quality assurance [20]. In a training hospital, such as this study’s institution, the use of VL may allow trainees safer intubation opportunities by direct visual supervision and coaching. Trainees who use a shared airway view with VL with their supervisors had higher intubation success rates [21]. They also reported that the teaching environment and constant guidance were helpful and improved their confidence [22].

There are several limitations to this study. The retrospective nature made accurate data collection challenging as intubation note detail varied between providers, including documentation of the number of attempts. JITT was performed with a convenience sample, during work hours, informally. As it was not tracked which providers participated in the JITT, it was not possible to determine if those who used VL were those who had JITT. The relatively small number of total intubations and subsequent AE made it challenging to draw specific conclusions, trends, and comparisons from the data. While VL use for pediatric intubations increased in the post-JITT period, causality cannot be attributed to the JITT as there may be other reasons use increased. However, the institution did have the VL device for three years and was not utilizing it with frequency, which was the occasion for the JITT implementation.

## Conclusions

The proportion of emergency pediatric intubations using VL increased after the institution of a JITT, especially in the ED. There was no significant change in IMAs and AEs. The infrequency of pediatric intubations makes drawing conclusions regarding the impact on IMAs and AEs challenging. Continued refresher training, especially during the COVID-19 pandemic where increased VL use is expected, maybe important to upskill providers and staff in the use of VL.
